# Association between triglyceride-glucose index, placental inflammatory cytokines, and perinatal outcomes in preeclampsia

**DOI:** 10.3389/fendo.2026.1878782

**Published:** 2026-09-16

**Authors:** Yuanyuan Mi, Jiaer Zhu, Ping Gong, Meijia He, Yurong Hua

**Affiliations:** Department of Obstetrics and Gynecology, Affiliated Hospital of Jiangnan University, Wuxi, Jiangsu, China

**Keywords:** inflammatory cytokines, perinatal outcomes, placenta, preeclampsia, triglyceride-glucose index

## Abstract

**Aim:**

To investigate the association between maternal TyG index and placental expression of the pro-inflammatory cytokines interleukin-6 (IL-6), tumor necrosis factor-alpha (TNF-α), and interleukin-1 beta (IL-1β) in women with preeclampsia, and to evaluate their relationship with perinatal outcomes.

**Methods:**

Pearson correlation analysis was used to examine associations between maternal TyG index and placental inflammatory cytokine expression, while exploratory ROC analysis was used to describe apparent in-sample discrimination of adverse perinatal outcome status.

**Results:**

The mean TyG index was 8.73 ± 0.41. Placental expression of IL-6, TNF-α, and IL-1β was observed in all specimens, with strong staining identified in 30.39%, 29.41%, and 26.47% of cases, respectively. TyG index was positively correlated with IL-6 (r = 0.41, p < 0.001), TNF-α (r = 0.34, p < 0.001), and IL-1β H-scores (r = 0.27, p = 0.008). In exploratory ROC analyses, the AUCs were 0.84 for TyG index, 0.79 for IL-6, 0.76 for TNF-α, and 0.73 for IL-1β. The exploratory combined model containing TyG index, IL-6, and TNF-α had an AUC of 0.88. These ROC estimates were not internally or externally validated.

**Conclusion:**

Maternal TyG index was associated with placental IL-6, TNF-α, and IL-1β expression among women with preeclampsia. Metabolic and inflammatory markers also showed apparent associations with adverse perinatal outcome status within this cohort. Because the study was observational, biomarkers were measured after diagnosis, placental markers were assessed after delivery, and the ROC findings were not validated, the results should not be interpreted as demonstrating causality, prospective prediction, or clinical utility.

## Introduction

Preeclampsia is a multifactorial pregnancy-specific disorder characterized by new-onset hypertension and end-organ dysfunction after 20 weeks of gestation. It remains a leading cause of maternal and perinatal morbidity and mortality worldwide. “Previous studies have reported associations between metabolic disturbances, particularly insulin resistance and dyslipidemia, and preeclampsia.The triglyceride-glucose (TyG) index, calculated from fasting triglyceride and glucose concentrations, has gained attention as a simple surrogate marker of insulin resistance and metabolic dysfunction. Previous studies have reported associations between elevated TyG index values during pregnancy and an increased risk of hypertensive disorders of pregnancy, including preeclampsia (1–5).

In addition to metabolic abnormalities, dysregulated inflammatory responses at the maternal–fetal interface are considered important contributors to placental dysfunction in preeclampsia. The maternal–fetal interface, composed of maternal decidual tissue and fetal trophoblasts, plays a critical role in maintaining immune tolerance and supporting normal placental development. Several investigations have reported altered expression of pro-inflammatory cytokines, including interleukin-6 (IL-6), tumor necrosis factor-alpha (TNF-α), and interleukin-1 beta (IL-1β), in placental tissues obtained from pregnancies complicated by preeclampsia (6–8). Previous studies have associated these cytokines with trophoblast invasion, vascular remodeling, endothelial function, and inflammatory signaling.

Increasing attention has been directed toward the potential interaction between metabolic dysfunction and placental inflammation. Insulin resistance has been associated with oxidative stress, lipotoxicity, endothelial dysfunction, altered adipokine activity, and inflammatory changes during pregnancy (9, 10). Consequently, the TyG index may reflect not only metabolic status but also broader pathophysiological processes linked to placental dysfunction. Previous studies have demonstrated positive associations between metabolic markers and inflammatory cytokines in pregnancy-related disorders, suggesting that metabolic and inflammatory pathways may act synergistically in the development and progression of preeclampsia (11–16).

Adverse perinatal outcomes, including low birth weight, preterm birth, low Apgar scores, neonatal intensive care unit (NICU) admission, and neonatal morbidity, occur more frequently in pregnancies complicated by preeclampsia. These outcomes are believed to result, at least in part, from impaired placental perfusion, chronic fetal hypoxia, and inflammatory-mediated placental injury. Understanding the relationship between maternal metabolic status and placental inflammatory activity may therefore provide important insights into factors associated with neonatal outcomes in affected pregnancies (17, 18). Although both the TyG index and placental inflammatory cytokines have been individually investigated in relation to preeclampsia, relatively few studies have explored their interrelationship within women affected by the disorder or examined their concurrent associations with perinatal outcomes. Most available studies have evaluated metabolic and inflammatory markers separately, limiting understanding of how these pathways may interact in the clinical manifestation of preeclampsia.

Therefore, the present study aimed to investigate the association between maternal TyG index and placental expression of IL-6, TNF-α, and IL-1β among women diagnosed with preeclampsia. We also examined the relationships of these metabolic and placental inflammatory markers with selected perinatal outcomes. The analyses were intended to characterize concurrent associations within the study cohort rather than to establish disease mechanisms, causality, prospective prediction, or clinical utility.

## Material and methods

### Study design and setting

This observational analytical study was conducted between January 2021 and January 2024 in the Departments of Obstetrics and Gynecology and Pathology at a tertiary care teaching hospital. The study aimed to investigate the association between the triglyceride–glucose (TyG) index and placental expression of inflammatory cytokines at the maternal–fetal interface among women diagnosed with preeclampsia and to evaluate their relationship with perinatal outcomes.

### Study population

A total of 102 consecutive pregnant women diagnosed with preeclampsia were recruited. The participant recruitment, eligibility assessment, and final inclusion process is summarized in **Figure 1**.

**Figure 1:**
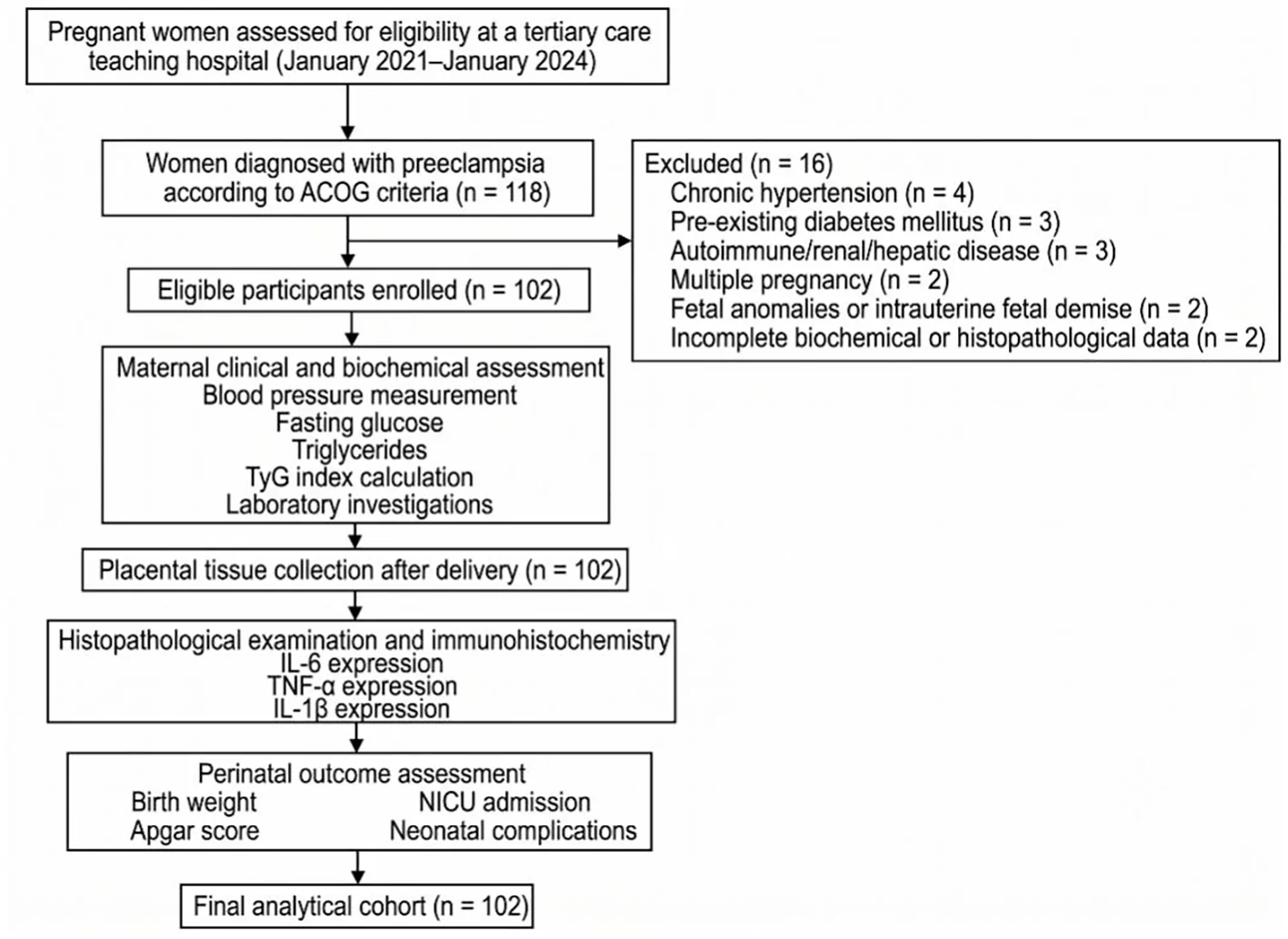
Flow diagram of participant recruitment, eligibility assessment, and final inclusion in the study. Flow diagram illustrating participant recruitment, eligibility assessment, exclusion criteria, placental tissue analysis, and inclusion of women with preeclampsia in the final study cohort (n = 102). Clinical, biochemical, immunohistochemical, and perinatal outcome assessments were performed for all included participants.

### Sample size and power considerations

No formal prospective sample-size calculation was used to determine participant recruitment. All consecutive women with preeclampsia who fulfilled the eligibility criteria during the predefined recruitment period were included, resulting in a final analytical cohort of 102 participants. During manuscript revision, a retrospective sensitivity analysis was conducted to describe the magnitude of correlation detectable with the available sample. At a two-sided alpha level of 0.05 and 80% statistical power, a sample of 102 participants could detect a Pearson correlation coefficient of approximately 0.27 or greater. This calculation represents a retrospective assessment of statistical sensitivity and was not used as an *a priori* basis for determining recruitment.

Preeclampsia was diagnosed according to the American College of Obstetricians and Gynecologists (ACOG) criteria as:

- Systolic blood pressure ≥140 mmHg and/or diastolic blood pressure ≥90 mmHg on two occasions at least 4 hours apart after 20 weeks of gestation, accompanied by
- Proteinuria ≥300 mg in a 24-hour urine collection or a urine protein-to-creatinine ratio ≥0.3.

### Inclusion criteria

- Maternal age 18–40 years
- Singleton pregnancy
- Gestational age ≥28 weeks
- Diagnosis of preeclampsia according to ACOG criteria
- Availability of placental tissue for histopathological analysis

### Exclusion criteria

- Chronic hypertension diagnosed before pregnancy
- Pre-existing type 1 or type 2 diabetes mellitus
- Chronic kidney disease
- Autoimmune disorders
- Chronic liver disease
- Multiple gestation
- Major fetal congenital anomalies
- Intrauterine fetal demise
- Current immunosuppressive therapy
- Incomplete clinical, biochemical, or histopathological records
- Gestational diabetes mellitus diagnosed during the index pregnancy

### Clinical and biochemical assessment

Demographic, obstetric, and clinical information were collected using a structured case record form. Variables included maternal age, body mass index (BMI), parity, gestational age at diagnosis and delivery, systolic and diastolic blood pressure, and presenting symptoms such as edema, headache, epigastric pain, and visual disturbances.

Fasting venous blood samples were collected within 24 hours of diagnosis. Fasting plasma glucose and serum triglyceride concentrations were measured using standardized enzymatic methods on an automated biochemical analyzer.

The TyG index was calculated using the following formula:


$$TyG\;=\;ln\;\left[fasting\;triglycerides\;\left(mg/dL\right)\;\times \;fasting\;glucose\;\left(mg/dL\right)/2\right]$$

Additional laboratory investigations included:

- Serum uric acid
- Serum creatinine
- Platelet count
- Alanine aminotransferase (ALT)
- Aspartate aminotransferase (AST)
- Lactate dehydrogenase (LDH)
- 24-hour urinary protein excretion

Fasting venous blood samples were collected within 24 hours of diagnosis and, whenever clinically feasible, before the initiation of antihypertensive medication, magnesium sulfate, or antenatal corticosteroid therapy. Treatment was not delayed for research-related blood collection in women requiring urgent clinical management. The timing of sampling relative to treatment administration was verified from the medical records.

### Placental tissue collection and immunohistochemistry

Placental tissue blocks were fixed in 10% neutral-buffered formalin, processed routinely, embedded in paraffin, and sectioned at a thickness of 4 μm. Sections were deparaffinized in xylene, rehydrated through graded ethanol, and subjected to heat-induced epitope retrieval in citrate buffer (pH 6.0) for 20 minutes. Endogenous peroxidase activity was blocked with 3% hydrogen peroxide for 10 minutes, followed by incubation with 5% normal goat serum for 20 minutes to reduce nonspecific staining. Representative hematoxylin and eosin (H&E) staining of placental tissue is shown in **Figure 2**.

**Figure 2:**
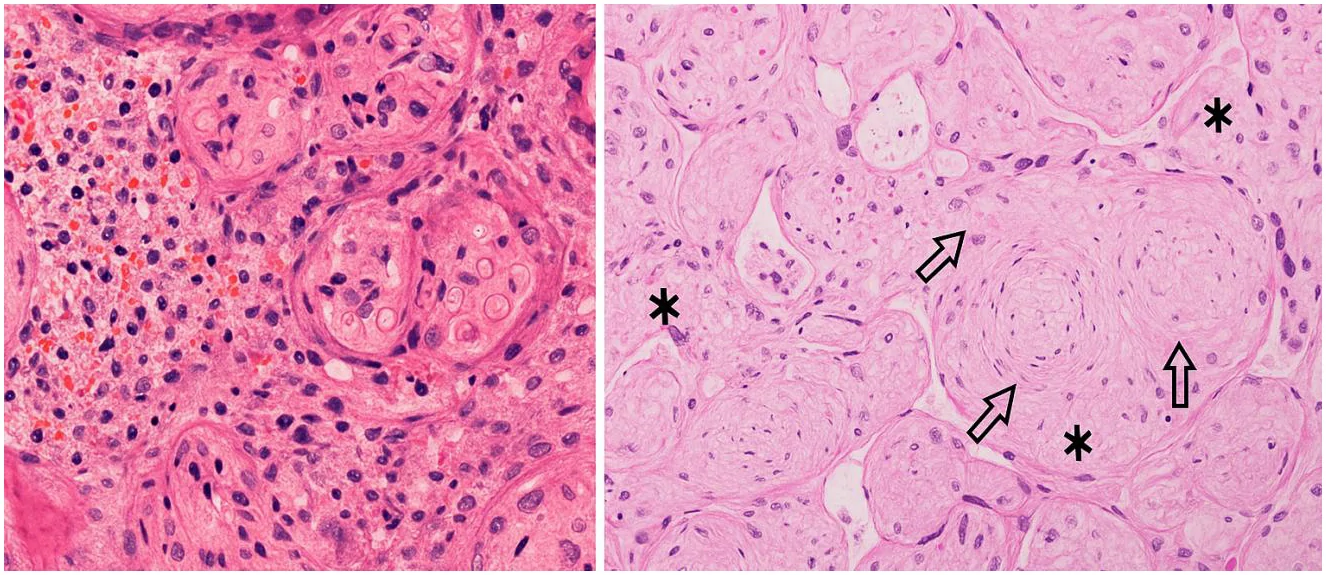
Hematoxylin and eosin (H&E) staining. * indicate areas of villous stromal fibrosis.

Additional data supporting the immunohistochemical procedures, including detailed information on primary antibodies used for placental tissue analysis, are available in **Supplementary Table S1**.

Primary antibodies were applied to tissue sections and incubated overnight at 4 °C in a humidified chamber. After washing with phosphate-buffered saline, sections were incubated with a horseradish peroxidase-labeled anti-rabbit polymer detection system for 30 minutes at room temperature. Immunoreactivity was visualized using 3,3′-diaminobenzidine chromogen, followed by hematoxylin counterstaining, dehydration, clearing, and mounting.

Human tonsil tissue containing inflammatory lymphoid and macrophage-rich areas was included as the positive control tissue for IL-6, TNF-α, and IL-1β staining in each staining batch. Negative controls were processed simultaneously by replacing the primary antibody with antibody diluent and, where available, a species- and isotype-matched normal rabbit immunoglobulin control at an equivalent protein concentration. Absence of specific brown cytoplasmic staining in the negative control sections was required for batch acceptance.

Positive staining was defined as brown cytoplasmic immunoreactivity in trophoblastic cells and villous stromal inflammatory cells. For each marker, five non-overlapping high-power fields were evaluated from representative areas of each placental section, avoiding infarcted, hemorrhagic, or necrotic tissue. At least 500 cells were assessed per case. Cytokine expression was quantified using the H-score method:

H-score = (1 × percentage of weakly stained cells) + (2 × percentage of moderately stained cells) + (3 × percentage of strongly stained cells), resulting in a score ranging from 0 to 300.

All slides were assigned anonymized study codes by a research assistant not involved in pathological scoring. The pathologist was blinded to maternal demographic details, clinical severity, biochemical parameters, TyG index, obstetric outcomes, and neonatal outcomes until completion of all IHC scoring.

For assessment of intra-observer reproducibility, 21 randomly selected cases, representing 20.6% of the cohort, were re-evaluated by the same pathologist after a two-week interval using newly randomized slide codes. Agreement between the initial and repeat H-score assessments was evaluated using a two-way mixed-effects intraclass correlation coefficient for absolute agreement. The observed ICC values and their 95% confidence intervals are reported in the Results section.

### Perinatal outcomes

The following perinatal outcomes were recorded:

- Gestational age at delivery
- Mode of delivery
- Birth weight
- Apgar scores at 1 and 5 minutes
- Umbilical cord pH (when available)
- NICU admission
- Respiratory distress syndrome
- Neonatal sepsis
- Neonatal hypoglycemia
- Neonatal mortality

For analytical purposes, an adverse perinatal outcome was defined as the occurrence of one or more of the following:

- Birth weight <2500 g
- Apgar score <7 at 5 minutes
- Umbilical cord pH <7.1
- Admission to the neonatal intensive care unit

Participants were subsequently categorized into adverse-outcome and non-adverse-outcome groups for exploratory ROC analysis and comparisons between explicitly defined clinical groups.

### Statistical analysis

Statistical analysis was performed using IBM SPSS Statistics version 26.0. Continuous variables were summarized as mean ± standard deviation, and categorical variables were reported as frequencies and percentages. Baseline maternal characteristics, biochemical measurements, placental staining distributions, and perinatal outcomes were summarized descriptively. No one-sample statistical tests against external population norms were performed.

Pearson correlation analysis was used to evaluate associations between maternal TyG index, placental IL-6, TNF-α and IL-1β H-scores, serum uric acid, and platelet count. Comparisons of adverse perinatal outcome frequencies between women with non-severe preeclampsia and preeclampsia with severe features, and between early-onset and late-onset preeclampsia, were performed using the chi-square test or Fisher’s exact test, as appropriate.

Exploratory receiver operating characteristic curve analysis was performed to assess the apparent ability of the TyG index and placental cytokine H-scores to discriminate between participants with and without the composite adverse perinatal outcome. For the combined analysis, predicted probabilities were obtained from a binary logistic model containing TyG index, IL-6 H-score, and TNF-α H-score, with composite adverse perinatal outcome status as the dependent variable. Areas under the curve and 95% confidence intervals were reported.

The ROC analyses were performed in the same cohort used to estimate the individual and combined curves. Calibration assessment, bootstrapping, cross-validation, external validation, and model-optimism correction were not performed. The ROC findings were therefore interpreted as exploratory, apparent in-sample discrimination and not as validated prospective predictive performance. All statistical tests were two-sided, and p < 0.05 was considered statistically significant.

Complete maternal TyG index, placental IL-6, TNF-α, and IL-1β H-score data were available for all 102 participants. Therefore, all 102 participants were included in each reported correlation analysis. The variables required to classify the composite adverse perinatal outcome were also available for all participants, and all 102 women were included in each individual and combined exploratory ROC analysis. No observations were excluded because of missing values, and no data imputation was performed.

## Results

All 102 participants had sufficient data to determine composite adverse perinatal outcome status and were included in each individual biomarker ROC analysis and in the combined TyG index, IL-6, and TNF-α model. No participants were excluded from the ROC analyses because of missing values.

### Baseline maternal characteristics

**Table 1** summarizes the baseline characteristics of the 102 women with preeclampsia. The mean maternal age was 29.4 ± 4.5 years, and the mean body mass index was 27.6 ± 3.2 kg/m². Sixty-eight participants (66.67%) were primigravida, and 34 (33.33%) were multigravida. The mean systolic and diastolic blood pressures were 152.3 ± 10.2 mmHg and 98.5 ± 8.4 mmHg, respectively. Edema was recorded in 76 participants (74.51%), headache or visual disturbances in 41 (40.20%), and visual blurring or flashes in 18 (17.65%). These findings are presented as descriptive characteristics of the enrolled cohort, and no comparisons with external population norms were performed.

**Table 1:** Baseline maternal characteristics of women with preeclampsia (N = 102).

Variable	Value
Maternal age (years)	29.4 ± 4.5
BMI (kg/m²)	27.6 ± 3.2
Gravidity	
Primigravida	68 (66.67%)
Multigravida	34 (33.33%)
Gestational age at diagnosis (weeks)	≥28 weeks
Systolic blood pressure (mmHg)	152.3 ± 10.2
Diastolic blood pressure (mmHg)	98.5 ± 8.4
Edema	76 (74.51%)
Headache/visual disturbances	41 (40.20%)
Visual blurring/flashes	18 (17.65%)

Data are presented as mean ± standard deviation or frequency (%).

### Biochemical parameters and TyG index distribution

The biochemical characteristics of the study cohort are presented in **Table 2**. The mean fasting glucose concentration was 92.1 ± 11.4 mg/dL, the mean triglyceride concentration was 178.6 ± 32.5 mg/dL, and the mean TyG index was 8.73 ± 0.41. Mean serum uric acid and creatinine concentrations were 6.1 ± 1.3 mg/dL and 0.89 ± 0.18 mg/dL, respectively, while the mean platelet count was 168.2 ± 42.7 × 10^9^/L. The mean ALT, AST, and LDH concentrations were 32.7 ± 10.5 U/L, 41.2 ± 12.9 U/L, and 488.2 ± 98.3 U/L, respectively. Mean 24-hour urinary protein excretion was 685.4 ± 215.6 mg/24 h. The remaining biochemical measurements are summarized in **Table 2**. These variables are reported descriptively, without statistical comparison against external reference values or population norms.

**Table 2:** Biochemical characteristics and TyG index among women with preeclampsia (N = 102).

Parameter	Mean ± SD
Fasting glucose (mg/dL)	92.1 ± 11.4
Triglycerides (mg/dL)	178.6 ± 32.5
TyG index	8.73 ± 0.41
Serum uric acid (mg/dL)	6.1 ± 1.3
Serum creatinine (mg/dL)	0.89 ± 0.18
Platelet count (×10^9^/L)	168.2 ± 42.7
ALT (U/L)	32.7 ± 10.5
AST (U/L)	41.2 ± 12.9
LDH (U/L)	488.2 ± 98.3
24-hour urine protein (mg/24 h)	685.4 ± 215.6
Hemoglobin (g/dL)	10.7 ± 1.1
Serum albumin (g/dL)	3.1 ± 0.5
Total cholesterol (mg/dL)	201.4 ± 38.7
LDL cholesterol (mg/dL)	121.9 ± 30.1
HDL cholesterol (mg/dL)	44.6 ± 8.2
Random blood sugar (mg/dL)	112.8 ± 16.2
Serum calcium (mg/dL)	8.4 ± 0.7
Serum magnesium (mg/dL)	1.6 ± 0.3
Total bilirubin (mg/dL)	0.9 ± 0.2

### Expression of inflammatory markers in placental tissue (N = 102)

The immunohistochemical expression of three important pro-inflammatory cytokines in placental tissue samples from preeclamptic women is shown in this table: interleukin-6 (IL-6), tumor necrosis factor-alpha (TNF-α), and interleukin-1 beta (IL-1β). The relative abundance of each cytokine at the maternal-fetal interface was represented by a semi-quantitative grading of its expression into weak (+), moderate (++), or strong (+++) staining patterns (**Table 3**; **Figures 3**–**5**). IL-6 was one of the inflammatory markers that showed high expression in every case. In particular, 30.39% (31/102) of the placentas had strong IL-6 expression, 48.04% (49/102) had moderate expression, and 21.57% (22/102) had weak IL-6 positivity. Immunohistochemical staining for IL-6, TNF-α, and IL-1β was identified in all 102 examined placental specimens, with variation in staining intensity across the cohort. For IL-6, weak staining was observed in 22 specimens (21.57%), moderate staining in 49 (48.04%), and strong staining in 31 (30.39%). For TNF-α, 25 specimens (24.51%) showed weak staining, 47 (46.08%) showed moderate staining, and 30 (29.41%) showed strong staining. For IL-1β, weak, moderate, and strong staining was observed in 30 (29.41%), 45 (44.12%), and 27 (26.47%) specimens, respectively.

**Table 3:** Placental expression of inflammatory cytokines (N = 102).

Marker	Weak (+)	Moderate (++)	Strong (+++)	100% expression
IL-6	22 (21.57%)	49 (48.04%)	31 (30.39%)	Yes
TNF-α	25 (24.51%)	47 (46.08%)	30 (29.41%)	Yes
IL-1β	30 (29.41%)	45 (44.12%)	27 (26.47%)	Yes

**Figure 3:**
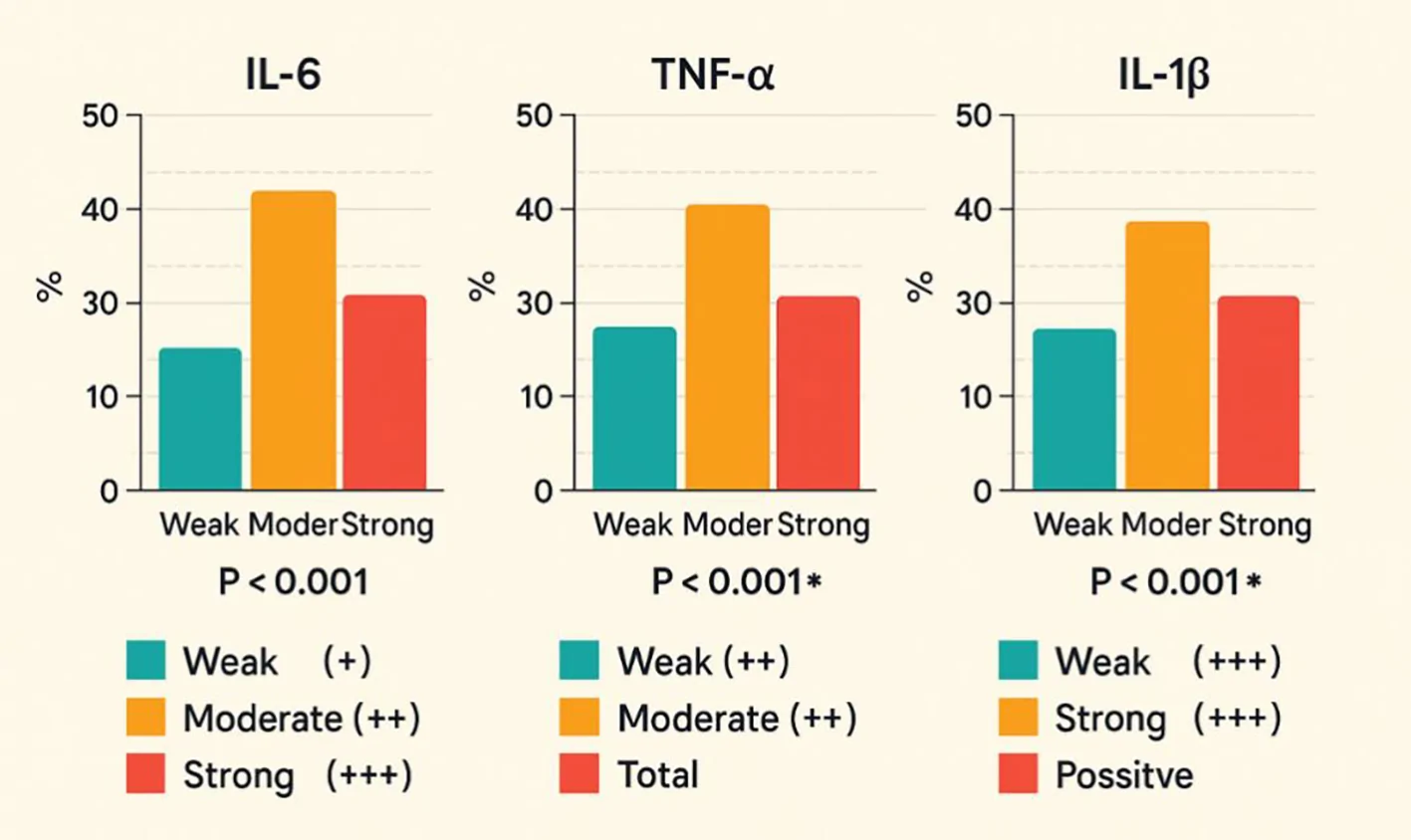
Expression of inflammatory markers in placental tissue (N = 102). * denotes statistical significance at P < 0.05.

**Figure 4:**
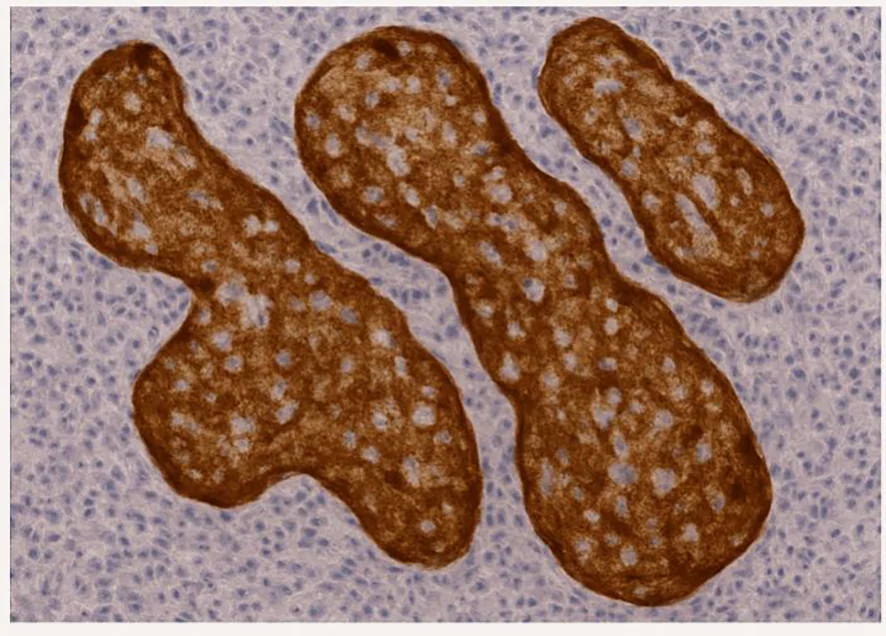
Histopathological image: strong IL-6 expression in placental tissue. This immunohistochemistry-stained section demonstrates intense (+++) cytoplasmic IL-6 expression (brown staining) in placental villi. The widespread and uniform staining pattern highlights strong local inflammatory activity in the placental microenvironment.

**Figure 5:**
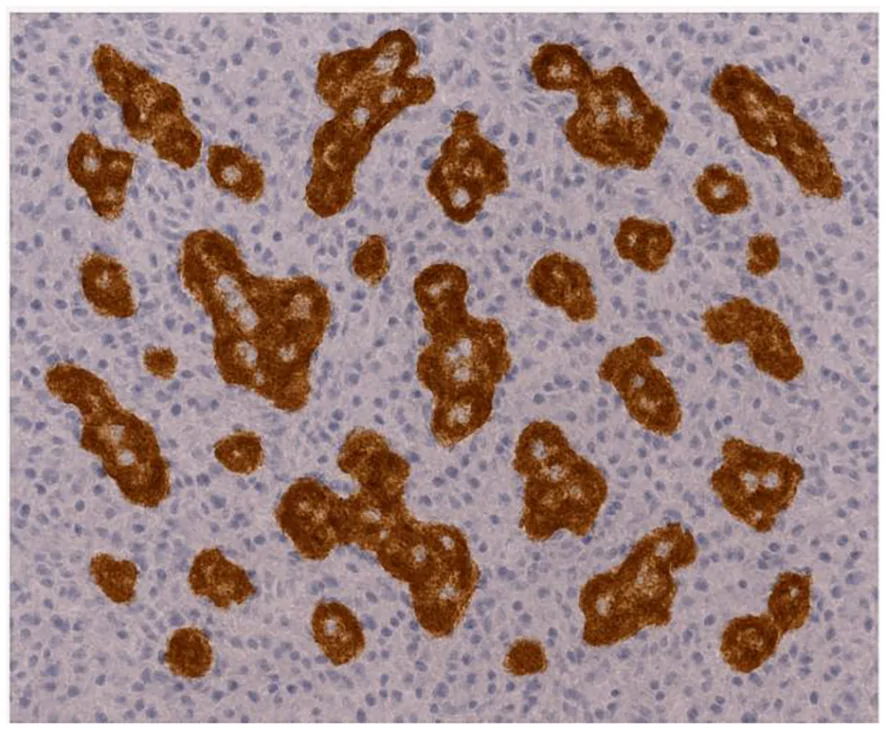
Histopathological image: strong TNF-α expression in placental tissue. This immunohistochemical section shows intense (+++) brown cytoplasmic staining for TNF-α within placental villi. The strong expression pattern reflects elevated local inflammation, indicative of immune activation in placental pathology.

These findings describe the distribution of cytokine staining among women with preeclampsia. Because no normotensive placental control group was included, the results do not establish that cytokine expression was elevated relative to uncomplicated pregnancies and should not be interpreted as evidence of a diagnostic, prognostic, or causal role.

Repeat scoring demonstrated good-to-excellent intra-observer agreement. The observed intraclass correlation coefficients were 0.91 for IL-6 (95% CI: 0.80–0.96), 0.88 for TNF-α (95% CI: 0.74–0.95), and 0.86 for IL-1β (95% CI: 0.70–0.94).

### Perinatal outcomes

Perinatal outcomes are summarized in **Table 4**. Cesarean delivery was performed in 73 pregnancies (71.57%). The mean gestational age at delivery was 35.1 ± 1.8 weeks, and the mean birth weight was 2320 ± 410 g. An Apgar score below 7 was recorded in 26 neonates (25.49%) at 1 minute and in 14 neonates (13.73%) at 5 minutes. Umbilical cord pH below 7.1 was recorded in 12 cases (11.76%), and 33 neonates (32.35%) were admitted to the NICU. Respiratory distress syndrome occurred in 10 neonates (9.80%), neonatal sepsis in 9 (8.82%), neonatal hypoglycemia in 10 (9.80%), and neonatal mortality in 5 (4.90%). These results describe the perinatal outcomes observed in the study cohort and were not compared with an external reference population.

**Table 4:** Perinatal outcomes.

Outcome	Value
Cesarean delivery	73 (71.57%)
Gestational age at delivery, weeks	35.1 ± 1.8
Birth weight, g	2320 ± 410
Apgar score <7 at 1 minute	26 (25.49%)
Apgar score <7 at 5 minutes	14 (13.73%)
Umbilical cord pH <7.1	12 (11.76%)
NICU admission	33 (32.35%)
Respiratory distress syndrome	10 (9.80%)
Neonatal sepsis	9 (8.82%)
Neonatal hypoglycemia	10 (9.80%)
Neonatal mortality	5 (4.90%)

Perinatal outcomes among women with preeclampsia (N = 102).

### Correlation between TyG index and placental inflammatory cytokine expression

Maternal TyG index was positively correlated with the placental IL-6 H-score (r = 0.41, p < 0.001), TNF-α H-score (r = 0.34, p < 0.001), and IL-1β H-score (r = 0.27, p = 0.008). A weaker positive correlation was observed between TyG index and serum uric acid (r = 0.18, p = 0.030), while an inverse correlation was observed between TyG index and platelet count (r = −0.22, p = 0.010). These results indicate statistical associations within the preeclampsia cohort and do not establish temporal or causal relationships (**Table 5**).

**Table 5:** Correlation between TyG index and placental inflammatory cytokine expression (N = 102).

Variable	Pearson correlation coefficient (r)	P-value
IL-6 H-score	0.41	<0.001*
TNF-α H-score	0.34	<0.001*
IL-1β H-score	0.27	0.008*
Serum uric acid	0.18	0.030*
Platelet count	-0.22	0.010*

Pearson correlation analysis was performed to evaluate associations between maternal TyG index and placental inflammatory cytokine expression. Positive coefficients indicate a direct relationship, whereas negative coefficients indicate an inverse relationship. P < 0.05 was considered statistically significant.

### ROC curve analysis of predictive biomarkers

Receiver Operating Characteristic (ROC) curve analysis is shown in **Table 6** and **Figures 6**, **7** to assess how well individual and combined biomarkers predict unfavorable perinatal outcomes in preeclamptic pregnancies. The diagnostic accuracy of each marker is indicated by the area under the curve (AUC), sensitivity, specificity, and p-values. With an AUC of 0.84 (95% CI: 0.77–0.91), the TyG index demonstrated a high degree of predictive power. This suggests that patients at risk for unfavorable neonatal outcomes can be identified with exceptional discriminatory power. Additionally, the TyG index correctly identified over 80% of neonates who were at risk and correctly ruled out over 75% of those who were not, with a sensitivity of 81.5% and a specificity of 78.2%. The outcome was statistically significant (P < 0.001), indicating that the TyG index, which measures metabolic dysfunction, is a strong and trustworthy early biomarker.

**Table 6:** Exploratory ROC analysis of TyG index and placental inflammatory markers for discrimination of composite adverse perinatal outcome status.

Marker	AUC (95% CI)	P-value
TyG index	0.84 (0.77–0.91)	<0.001
IL-6 H-score	0.79 (0.71–0.87)	<0.001
TNF-α H-score	0.76 (0.68–0.85)	0.006
IL-1β H-score	0.73 (0.64–0.82)	0.009
Combined model: TyG index + IL-6 H-score + TNF-α H-score	0.88 (0.82–0.94)	<0.001

Receiver operating characteristic analyses were performed as exploratory assessments of apparent discrimination between participants with and without a composite adverse perinatal outcome. The composite outcome was defined as one or more of the following: birth weight <2500 g, Apgar score <7 at 5 minutes, umbilical cord pH <7.1, or NICU admission. Predicted probabilities from a binary logistic model containing TyG index, IL-6 H-score, and TNF-α H-score were used for the combined ROC curve. The analyses were conducted in the same cohort used to estimate the curves. Calibration, bootstrapping, cross-validation, external validation, and optimism correction were not performed. AUC, area under the curve; CI, confidence interval.

**Figure 6:**
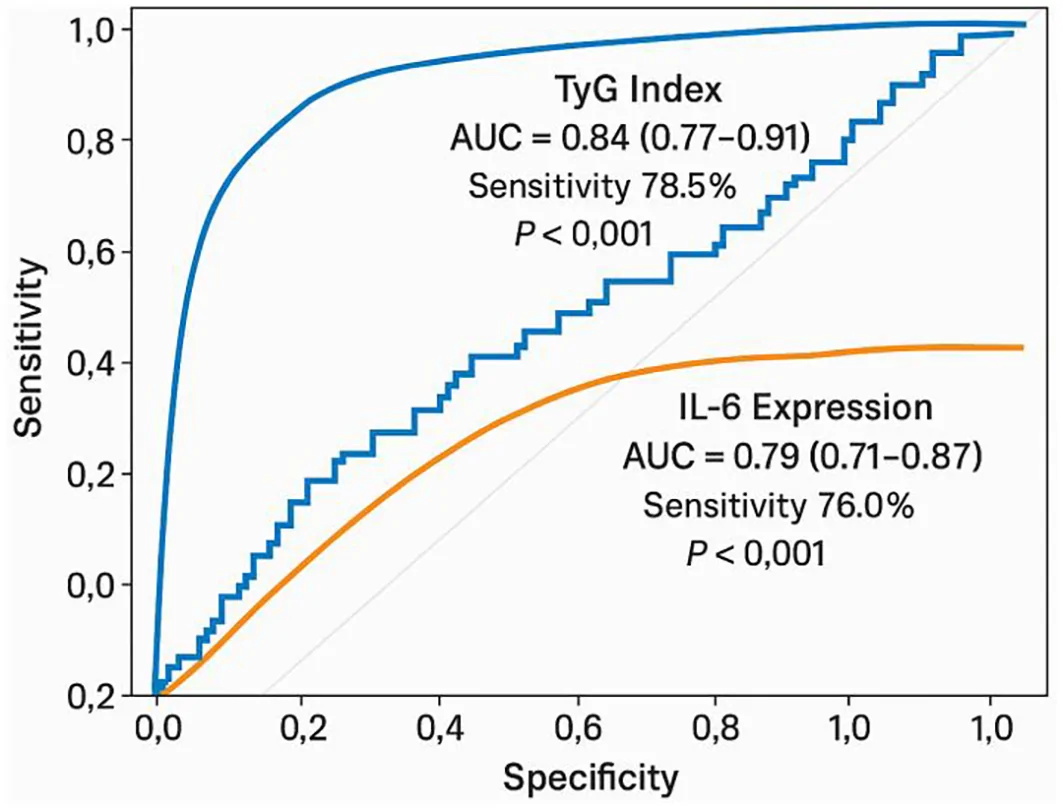
Exploratory ROC curves for individual metabolic and placental inflammatory markers.

**Figure 7:**
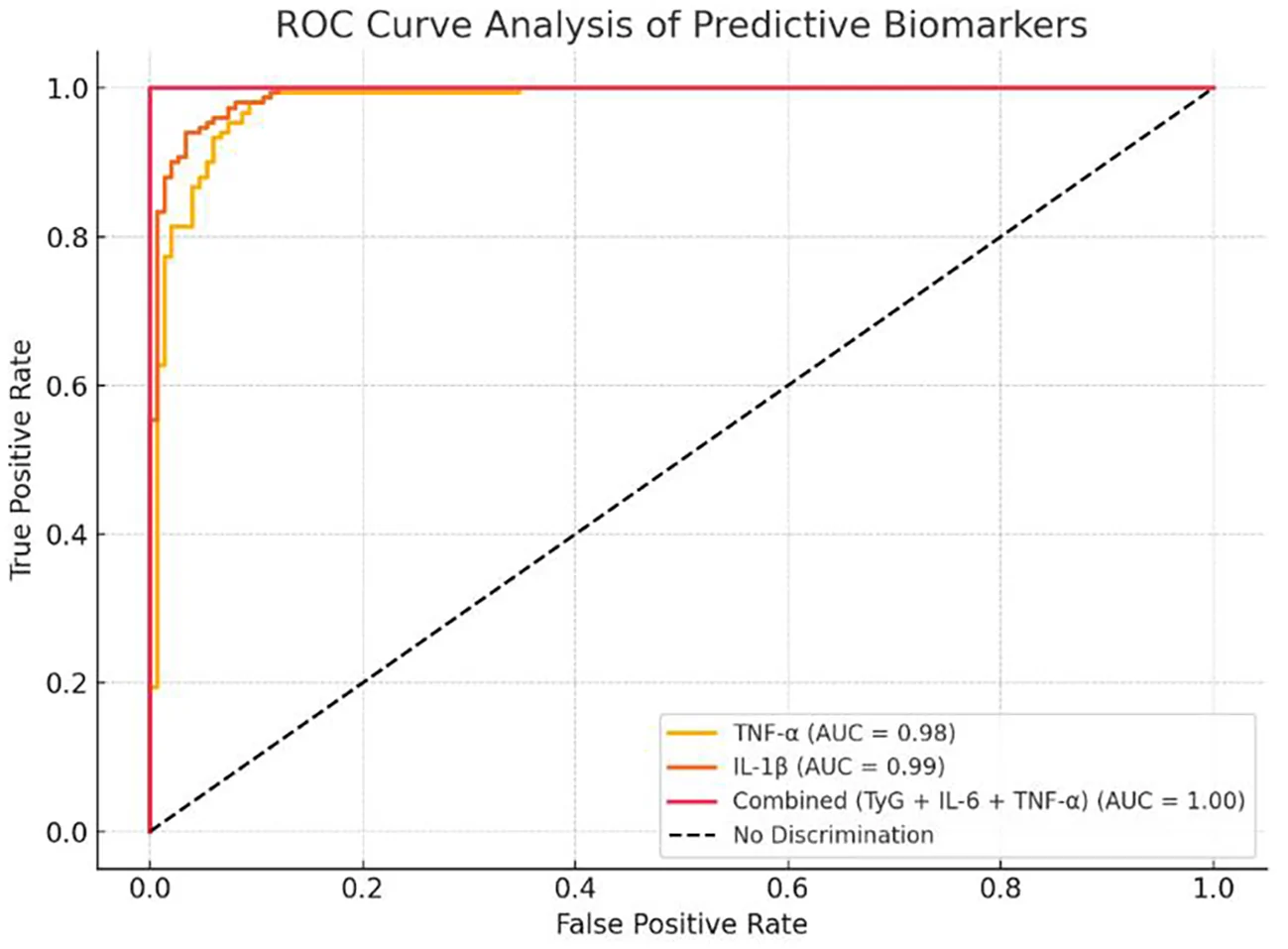
Exploratory ROC curve for the combined TyG index, IL-6, and TNF-α model.

With an AUC of 0.79 (CI: 0.71–0.87), sensitivity of 76.0%, and specificity of 74.5%, IL-6 expression—a measure of systemic and placental inflammation—also showed excellent predictive accuracy. This statistically significant result (P < 0.001) supports IL-6 as a key immunological biomarker in preeclampsia-related morbidity and reaffirms the role of inflammatory pathways in determining fetal compromise.

With an AUC of 0.76 (CI: 0.68–0.85), sensitivity of 72.5%, and specificity of 70.3%, TNF-α expression also demonstrated strong predictive value. The prediction strength was significant (P = 0.006) despite being marginally lower than that of TyG and IL-6, highlighting the involvement of TNF-α in vascular pathology and placental inflammation. With an AUC of 0.73 (CI: 0.64–0.82), sensitivity of 68.0%, and specificity of 67.1%, IL-1β expression also demonstrated a respectable diagnostic performance. Despite having the lowest AUC of all the individual markers, it was still a statistically significant predictor (P = 0.009), indicating that it played a role in negative outcomes, albeit a less significant one. With a remarkable sensitivity of 84.3% and specificity of 79.1%, the combined model (TyG + IL-6 + TNF-α) produced the highest AUC of 0.88 (CI: 0.82–0.94). This finding was statistically very significant (P < 0.001) and suggests that integrating metabolic and inflammatory markers provides the most accurate risk stratification for predicting neonatal complications in preeclamptic pregnancies (**Table 6**).

Among the 102 women with preeclampsia, 59 (57.84%) had preeclampsia with severe features and 43 (42.16%) had non-severe disease. Adverse perinatal outcomes occurred more frequently in women with severe disease than in those with non-severe preeclampsia (71.19% vs. 32.56%, *p* < 0.001). Early-onset preeclampsia (<34 weeks) was observed in 35 women (34.31%), whereas 67 women (65.69%) had late-onset preeclampsia. Adverse perinatal outcomes were significantly more common in early-onset than late-onset preeclampsia (80.00% vs. 41.79%, *p* < 0.001) (**Table 7**).

**Table 7:** Distribution of preeclampsia severity, timing of onset, and adverse perinatal outcomes (N = 102).

Variable	Total, n (%)	Adverse perinatal outcome, n (%)	P value
Severity of preeclampsia			
Non-severe preeclampsia	43 (42.16%)	14 (32.56%)	
Preeclampsia with severe features	59 (57.84%)	42 (71.19%)	<0.001
Timing of onset			
Early-onset preeclampsia (<34 weeks)	35 (34.31%)	28 (80.00%)	
Late-onset preeclampsia (≥34 weeks)	67 (65.69%)	28 (41.79%)	<0.001

## Discussion

The present study examined the associations between maternal TyG index, placental inflammatory cytokine expression, and perinatal outcomes among women diagnosed with preeclampsia. Maternal TyG index was positively correlated with placental IL-6, TNF-α, and IL-1β H-scores. Adverse perinatal outcomes were also observed frequently within the cohort. These findings indicate that metabolic and inflammatory abnormalities co-occurred in women with established preeclampsia but do not establish temporal, mechanistic, or causal relationships. The demographic and biochemical characteristics of the cohort were generally consistent with clinical features previously reported among women with preeclampsia. Most participants were primigravida, and the mean BMI was 27.6 kg/m². Previous studies have reported associations of maternal adiposity, insulin resistance, oxidative stress, and endothelial dysfunction with hypertensive disorders of pregnancy. However, because the present study did not include a normotensive comparison group, the measured biochemical values cannot be interpreted as elevated or reduced relative to uncomplicated pregnancies. They should be regarded as descriptive characteristics of the enrolled cohort. The mean TyG index was 8.73, and positive correlations were observed between the TyG index and placental IL-6, TNF-α, and IL-1β H-scores. Previous studies have also reported relationships between metabolic indices and inflammatory markers in pregnancy-related disorders (19–23). The present findings are compatible with an association between maternal metabolic status and placental inflammatory activity in established preeclampsia (24–26). Nevertheless, the cross-sectional nature of the measurements does not establish whether one abnormality preceded, influenced, or mediated the other.

Placental staining for IL-6, TNF-α, and IL-1β was identified in all examined specimens, with variation in staining intensity. These cytokines have previously been studied in relation to trophoblast function, vascular remodeling, endothelial activation, and inflammatory signaling (27–30). In the present study, however, the absence of placental samples from normotensive pregnancies prevents conclusions regarding disease-specific overexpression. The staining findings therefore describe variation within women with preeclampsia and do not demonstrate that these cytokines caused placental dysfunction or disease progression (31–34).

The cohort experienced frequent preterm delivery, low birth weight, low Apgar scores, umbilical cord acidosis, and NICU admission. Women with preeclampsia with severe features and those with early-onset disease had higher frequencies of the composite adverse perinatal outcome than women with non-severe or late-onset disease (35–37). These findings represent clinical associations within the cohort. They do not establish that the measured metabolic or placental inflammatory markers directly caused the observed neonatal outcomes. Exploratory ROC analyses yielded AUC values ranging from 0.73 to 0.84 for individual markers and an AUC of 0.88 for the combined TyG, IL-6, and TNF-α model. These results describe apparent discrimination within the same cohort used to develop and assess the curves (38–42). They should not be interpreted as validated predictive performance because calibration, bootstrapping, cross-validation, external validation, and correction for model optimism were not performed. Furthermore, placental cytokines were assessed after delivery and therefore cannot support antenatal prediction or early clinical risk stratification in the present study. Overall, the observed correlations are consistent with the coexistence of metabolic and inflammatory abnormalities in established preeclampsia. They provide a basis for further investigation but do not demonstrate a metabolic–inflammatory mechanism, biological mediation, disease progression, or clinical utility. Prospective multicenter studies with longitudinal measurements and appropriate comparison groups are required to clarify the temporal and biological relationships among these variables (43–46).

Several limitations should be considered. First, the study was conducted at a single tertiary-care center and included a relatively modest sample, which may limit generalizability. Second, there was no normotensive control group, preventing direct comparison of metabolic and placental inflammatory markers with uncomplicated pregnancies. Third, the observational design does not permit causal inference. Fourth, placental cytokines were measured after delivery and reflect established disease rather than early pregnancy changes. Fifth, the ROC analyses were exploratory and lacked calibration assessment, internal validation, external validation, and optimism correction. Sixth, immunohistochemical evaluation was semi-quantitative, although repeat scoring demonstrated good-to-excellent intra-observer agreement. Finally, neonatal follow-up was limited to immediate perinatal outcomes.

Future prospective, multicenter studies should measure metabolic and inflammatory markers before the development of clinical outcomes, include appropriate normotensive comparison groups, and use prespecified modeling strategies with calibration and internal and external validation. Such studies are needed before any conclusions can be made regarding early prediction, clinical risk stratification, or implementation of combined metabolic and inflammatory biomarkers.

## Conclusion

This study identified positive associations between maternal TyG index and placental IL-6, TNF-α, and IL-1β expression among women with preeclampsia. Metabolic and placental inflammatory markers were also associated with adverse perinatal outcome status within the study cohort. These findings are consistent with the coexistence of metabolic and inflammatory abnormalities in established preeclampsia. However, the observational cross-sectional design, absence of a normotensive comparison group, post-diagnosis measurement of maternal biomarkers, post-delivery assessment of placental cytokines, and lack of ROC validation prevent conclusions regarding causality, disease mechanisms, prospective prediction, or clinical utility.

These findings need to be understood in the context of several limitations. First, the study was not accompanied by a normotensive control group. All participants were women who had already developed preeclampsia, and results only represent associations and biomarker variability among these women who developed preeclampsia. The study is unable to establish whether or not the concentration of IL-6, TNF-α and IL-1β in the placenta was higher, lower or not significantly different in the pregnancies that were complicated by preterm delivery compared to pregnancies without such complications. All of the placental specimens should therefore not necessarily be considered to have evidence of disease-specific overexpression based on detection of cytokine staining. Second, this study was carried out at one tertiary care center and with a fairly small sample size which may restrict the generalizability of the results. Third, lacks causal and temporal inferences due to the type of design (observational and cross-sectional design). Fourth, the maternal metabolic biomarkers were analyzed following the diagnosis of preeclampsia; placental cytokine expression was measured after delivery; hence, these measurements cannot establish early pathogenic changes or support prospective antenatal prediction. Fifth, exploratory ROC analyses were performed in the same cohort as that for curve estimation, and were not evaluated for calibration, internal validation, external validation, or model optimism. Sixth, immunohistochemical evaluation was semi-quantitative, but repeat scoring showed good to excellent intra-observer agreement. Finally, assessment of the infants was limited to immediate perinatal outcomes and long-term infant follow-up was not available.

## Data Availability

The original contributions presented in the study are included in the article/**Supplementary Material**. Further inquiries can be directed to the corresponding author.

## Supplementary material

Supplementary Table 1 Primary antibodies used for immunohistochemical analysis of placental tissue.
