# Supplementary material for: Association between triglyceride-glucose index, placental inflammatory cytokines, and perinatal outcomes in preeclampsia
Source: Front Endocrinol (Lausanne). 2026 Sep 16;17:1878782. doi: 10.3389/fendo.2026.1878782 (PMC13623745; doi:10.3389/fendo.2026.1878782)
Supplement: Supplementary file 1 [file Table1.docx]

**Supplementary Table S1. Primary Antibodies Used for Immunohistochemical Analysis of Placental Tissue**

| **Target** | **Primary antibody** | **Manufacturer** | **Catalogue no.** | **Host / clonality** | **Species / IHC application** | **Working dilution used** | **Antigen retrieval / incubation** | **Positive control** |
| --- | --- | --- | --- | --- | --- | --- | --- | --- |
| IL-6 | Anti-IL-6 antibody | Abcam | ab6672 | Rabbit polyclonal | Human; IHC-P | 1:400 | Citrate buffer, pH 6.0, heat-induced retrieval for 20 min; overnight at 4°C | Human tonsil tissue |
| TNF-α | Anti-TNF alpha antibody | Abcam | ab6671 | Rabbit polyclonal (IgG) | Human; IHC-P | 1:200 | Citrate buffer, pH 6.0, heat-induced retrieval for 20 min; overnight at 4°C | Human tonsil tissue |
| IL-1β | Anti-IL-1 beta antibody | Abcam | ab2105 | Rabbit polyclonal (IgG) | Human; IHC-P | 1:20 | Citrate buffer, pH 6.0, heat-induced retrieval for 20 min; overnight at 4°C | Human tonsil tissue |

**IHC procedure and controls.** Placental blocks were fixed in 10% neutral-buffered formalin, paraffin embedded, and sectioned. After deparaffinization and antigen retrieval, endogenous peroxidase was blocked with 3% hydrogen peroxide for 10 min and nonspecific binding with 5% normal goat serum for 20 min. After primary-antibody incubation, an HRP-labeled anti-rabbit polymer detection system was applied for 30 min at room temperature, followed by DAB visualization and hematoxylin counterstaining. Negative controls omitted the primary antibody.

**Abbreviations:** DAB, 3,3′-diaminobenzidine; HRP, horseradish peroxidase; IHC-P, immunohistochemistry on formalin-fixed paraffin-embedded tissue; IL, interleukin; TNF-α, tumor necrosis factor alpha.
